# Can a biologist fix a smartphone?—Just hack it!

**DOI:** 10.1186/s12915-017-0378-2

**Published:** 2017-05-08

**Authors:** Sophien Kamoun

**Affiliations:** The Sainsbury Laboratory, Norwich Research Park, Norwich, NR4 7UH UK

## Abstract

Biological systems integrate multiscale processes and networks and are, therefore, viewed as difficult to dissect. However, because of the clear-cut separation between the software code (the information encoded in the genome sequence) and hardware (organism), genome editors can operate as software engineers to hack biological systems without any particularly deep understanding of the complexity of the systems.

This article was inspired by the influential and entertaining essay by Yuri Lazebnik who argued that there are fundamental flaws in how biologists approach problems [1]. Lazebnik proposed that the complexity of biological systems calls for a systems approach to the study of living systems using a radio as a colourful metaphor to illustrate his points [1]. He postulated that, conceptually, a radio functions similarly to a biological system by converting a signal from one form into another using a signal transduction pathway [1]. Here I argue that Lazebnik’s thesis is limited by two fundamental principles of biology. First, the clear-cut separation between the software code—the operating information for living systems as written in the genome sequence—and hardware, or the organism itself [2, 3]. Second, biological systems are not optimally designed but are shaped by historicity—the historical constraints that are integral to their evolution [4]. This limits the extent to which principles of design and engineering can be useful in understanding and manipulating the structures and functions of living organisms. In contrast, modern day biologists are starting to operate as software engineers to hack biological systems and write apps despite a somewhat superficial understanding of the underlying complexity of these systems.

## How to fix a smartphone

Let me build on Lazebnik’s metaphor but instead of biologists, I’ll have aliens from a technologically advanced civilization visit earth and study man-made objects. And rather than a radio, I use a smartphone as the metaphor for living organisms because of the unambiguous separation between the codical domain (information coded in the software) and material domain (hardware)—a fundamental concept that defines all biological systems [2, 3].

Our visitors, which we’ll assume are generously funded to perform basic exploratory research by limitless grants from the Alien Science Foundation, become intrigued by smartphones, which they note are ubiquitously used by humans throughout their daily routines. They pry open a phone, and not only do they describe and classify the shape, color, and size of every component but they also measure all electronic connections and signals using the most sophisticated technology. They recruit human users to play with the phone and study how the phone responds to the user’s actions. They achieve a perfect understanding of the system dynamics at multiple levels from a single electronic chip to the phone in its entirety to the way the phone responds to its user’s actions. They develop a perfect understanding of the phone in all its intricate physical and electronic details. They write complex mathematical models that describe how the phone operates and responds to the user’s stimuli. They even figure out how individual phones interact with each other and how they form higher-level webs of complex and dynamic networks ruled by various laws and principles.

**Fig. 1.**
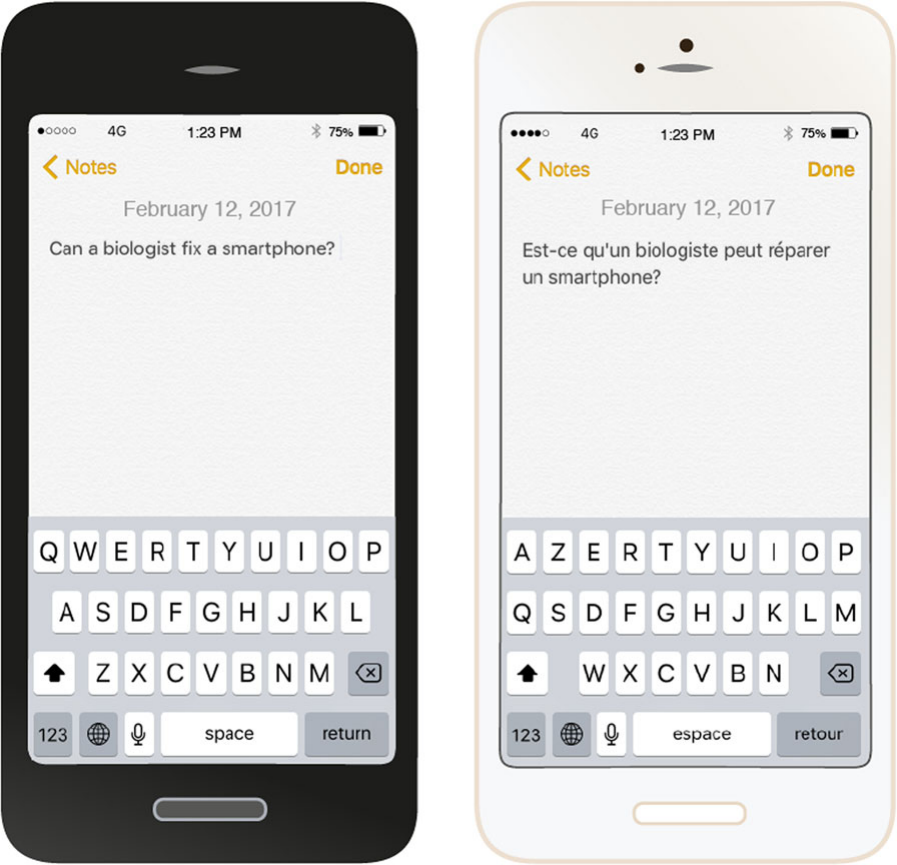
The QWERTY and AZERTY keyboards of smartphones

Yet despite this ultimate sophisticated understanding of the system, our aliens will not figure out why the letters in the phone keyboards are arranged in the QWERTY configuration (Fig. 1) [5]. This is because a smartphone fulfils another fundamental feature of living organisms—historicity, the historical constraints that have shaped its evolution [4, 5]. The QWERTY keyboard in a smartphone is a historical artefact from an early keyboard design that became widely adopted because it was integrated in one of the first commercial typewriters [5, 6]. There is no particular need for such a keyboard arrangement in modern smartphones and computers, and our aliens will be hard pressed to identify the raison d'être of the QWERTY keyboard by studying modern human societies.

This example illustrates the limits of systems approaches to studying living organisms. Organisms are not optimally designed. They are messy. They have evolved through evolutionary tinkering [4, 7, 8]. They suffer from the constraints of being linked through the unbroken chain of heredity to a series of ever more distant ancestors. This fundamental concept is what most vividly differentiates biology from design and engineering.

How would our aliens crack the problem? Which approach would allow them to gain useful insights into the QWERTY question? The breakthrough will emerge from studying and comparing multiple phones. Let’s say our aliens have landed in multicultural London. Rather than studying and dissecting a single phone, they end up collecting a whole bunch of them. A persistent young alien scientist named Karla Darwin noticed after long hours of careful observation that letters in some keyboards are arranged differently. Whereas most phones have their keys organized as QWERTY, others follow the AZERTY configuration (Fig. 1). Young Karla is intrigued by this odd finding and spends much time researching why this is the case. At some point, and after many fruitless leads, Karla discovers a striking correlation between the keyboard organization and the language used by the phone’s users. She collects more comparative data between QWERTY and AZERTY phones, and performs experiments such as swapping keyboards and then noting that the users initially type gibberish but can still switch back to typing correctly although with reduced speed. She discovers that keyboards are not unique to smartphones but are found in other devices, some of which are no longer in use. A breakthrough occurs when on one of her random London walks she discovers typewriters in an antique shop on Portobello Road and realizes that these have relic keyboards that predate computers and smartphones. She gains much insight from this comparative analysis, which allows her to develop and test various hypotheses on the ancestral functions of the keyboard. After many years of systematic analyses, she develops the theory that keyboards have evolved from a common extinct form and have diverged into different patterns to adapt to the user’s language. She speculates, although she struggles to fully demonstrate this, that the various keyboard designs were probably useful in some way in the relic typewriter keyboard. She stands accused of adaptationism by some of her colleagues, who ridicule her with extreme examples. Nonetheless, Karla Darwin persists and her treatise “On The Origin Of Keyboards” will become widely viewed as the birth of the alien science of evolution.

Here lies the limit of Lazebnik’s thesis that there are fundamental flaws in how biologists approach problems. Astute biologists would not study just one single model radio or phone (although many do). They will study populations of radios and phones that vary so slightly yet yield different phenotypes shaped by their descent from a common ancestral form. Some of these changes will be functional (adaptive), explaining differences in how the radio or phone operates. Others would be the product of chance. Yet, many differences will be the products of historical constraints—vestiges of past adaptations or random events that have drifted over time into endless shapes and patterns that sharply deviate from the rules of design. This is why, in the absence of an evolutionary perspective, understanding the systems features of the hardware in all their intricate details is unlikely to shed light on the innate messiness of biological systems.

## Hacking phones. Hacking genomes

To fix biological systems we need to focus on the codical domain. Think of the smartphone. What is more powerful? What makes the biggest difference in the outputs the system can achieve? Is it changes to the phone’s hardware—which require a deep and interconnected understanding of the components and electronics. Or is it changes to the software? Programmers only need to understand how to interact with the existing software in the system to create new apps of boundless creativity and sophistication. Surely both hardware and software engineers are needed. But the software engineer has so much more power to create astounding new functions in the system.

The capacity to read and edit the genetic language encoded in the genome has enabled biologists to access the codical domain of living systems in an unprecedented fashion. Biologists have started to operate as software engineers who can hack and write applications to produce the most original outputs. Rewriting the language of life and manipulating the codical domain of biological systems doesn’t necessitate a full understanding of the material complexity of living organisms. However, an evolutionary perspective developed through comparative genetic and functional analyses allows biologists to make educated guesses on how to modify and retool the code.

Genome editing is a new evolutionary force. It accelerates a process that has taken place for eons. Instead of studying living organisms in isolation or independently of their genetic code, we need to fully integrate evolutionary analyses in all aspects of biological research [4]. The true power of genome editing will only become evident when we comprehensively understand the unique tinkering nature of the evolutionary process, and the trade-offs that constrain it. This combination of genome editing with better appreciation of the centrality of evolution promises to revolutionize basic and applied biology and cement the position of biology as an information science.
